# Ocular abnormalities in congenital Zika syndrome: a case report, and review of the literature

**DOI:** 10.1186/s13256-018-1679-y

**Published:** 2018-06-09

**Authors:** Jade Gieseke Guevara, Swati Agarwal-Sinha

**Affiliations:** 1 Department of Ophthalmology, University of Florida, Gainesville, FL USA; 2 Department of Ophthalmology, Retinopathy of Prematurity Services, University of Florida, Gainesville, FL, 1600 SW Archer Road, Gainesville, FL 32610 USA

**Keywords:** Ocular abnormalities, Macular coloboma, Congenital Zika syndrome, Microcephaly

## Abstract

**Background:**

As the number of children with Zika virus-related complications grows, the long-term developmental trajectory and its effects on families are unknown. We present the first known case of congenital Zika syndrome seen at our institution with significant fundus findings.

**Case presentation:**

A 3-day-old Hispanic baby girl presented with severe microcephaly of 24 cm and temperature instability at birth. Her mother had traveled to Honduras early in pregnancy and testing of amniotic fluid was positive for Zika virus via polymerase chain reaction. A dilated fundus examination was significant for bilateral severe colobomatous chorioretinal atrophy of the macula and pigmentary changes. Neonatal magnetic resonance imaging revealed diffuse lissencephaly with decreased brain volume, atrophic corpus callosum and brainstem, periventricular calcifications, and ventriculomegaly of the lateral ventricles.

**Conclusions:**

Our patient, who presented with the first known case of congenital Zika syndrome in Northern Florida, demonstrated profound bilateral colobomatous chorioretinal atrophy of the macula. The ophthalmologic findings along with severe microcephaly emphasize the neurotropism of the Zika virus, and ultimately are indicative of poor developmental and visual prognosis for affected infants. With the increased prevalence of Zika virus, ophthalmologists should be aware of the associated findings and the importance of an eye-screening examination with a dilated fundus examination within 1 month of life of infants in which congenital Zika syndrome is suspected. A multidisciplinary care approach is essential for the care of affected infants and their families.

## Background

The Zika virus (ZKV) epidemic is a major public health issue that has received increasing attention since the World Health Organization declared a global public health emergency in February 2016 [1]. ZKV has spread to almost every country in the Western hemisphere except Canada [2]. Infants exposed to ZKV *in utero* have presented with severe central nervous system (CNS) defects, with microcephaly the most commonly reported [3]. The constellation of abnormalities associated with the virus is described as congenital Zika syndrome (CZS). Reports in the literature have described vision-limiting retina and optic nerve abnormalities such as atrophic retina, pigmentary changes, and optic nerve pallor in infants [4–8].

## Case presentation

Ophthalmologic consultation at the University of Florida in Gainesville was requested for a 3-day-old Hispanic baby girl. She was born at 36 weeks via induced vaginal delivery with features of severe CZS. The infant’s mother had traveled to Honduras early in pregnancy prior to knowing she was pregnant. Prenatal magnetic resonance imaging (MRI) revealed prominent ventricles, microcephaly, cerebral, cerebellar, and brainstem atrophy. Amniotic fluid via amniocentesis tested positive for Zika serologies. Following birth, the infant presented with slow respirations, bradycardia, and temperature instability. She stabilized and was able to breastfeed and demonstrated improvement of her vital signs. On physical examination, she was noted to have bilateral talipes equinovares and arthrogryposis.

**Fig. 1:**
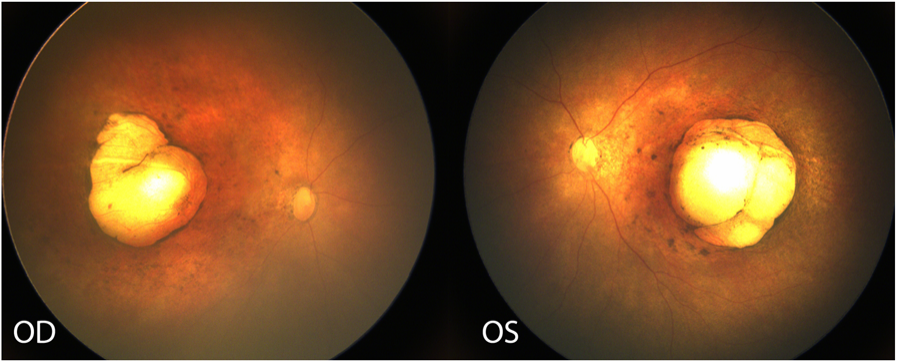
Color fundus picture of the right (oculus dextrus) and left (oculus sinister) eye of 3-day old baby girl with congenital Zika syndrome with bilateral macular colobomatous like chorioretinal atrophy, attenuated vessels, pigmentary changes, and optic disc pallor

**Fig. 2:**
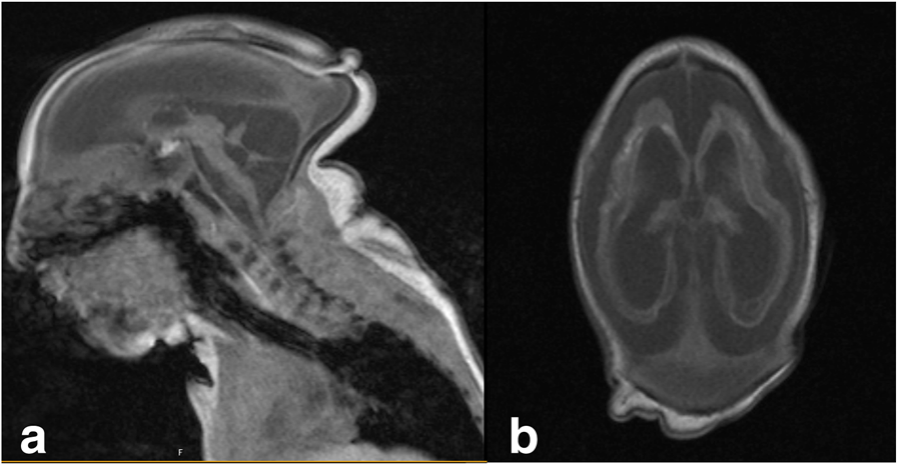
**a** Neonatal brain magnetic resonance imaging T1-weighted image demonstrating grossly abnormal brain formation with diminished brain volume and diffuse lissencephaly in the supratentorial brain parenchyma. There is marked atrophy of the corpus callosum, cerebellum, and brainstem. **b** Neonatal brain magnetic resonance imaging T1-weighted image highlighting ventriculomegaly, increased T1 signal in the cortical-subcortical white matter junction at areas of dystrophic calcification with diffuse ventriculomegaly

On external examination, her head circumference was 24.0 cm, which is categorized as severe microcephaly. She was noted to blink to light and her globes were soft to palpation bilaterally. An anterior segment examination was unremarkable and her corneal diameter was noted to be 9.5 mm bilaterally. A dilated fundus examination was significant for a large, well-circumscribed area of colobomatous-like excavation with chorioretinal atrophy in the bilateral maculae. The lesions were noted to have a hyperpigmented border and scleral show (Fig. 1). Additionally, optic disc pallor, vessel attenuation, and retinal pigmentary changes were noted in both eyes. Other communicable diseases including hepatitis B, human immunodeficiency virus (HIV), syphilis, toxoplasmosis, rubella, and cytomegalovirus were ruled out. The infant’s newborn screen and cytogenetics panel returned within normal limits. A neonatal brain MRI at 4 days of life revealed severe microcephaly and lissencephaly, diffuse atrophy of the corpus callosum and cerebellum, dystrophic calcifications, and marked thinning of the basal ganglia and brainstem (Fig. 2a, b). Additionally, ventriculomegaly was noted in all ventricles.

The infant remained hemodynamically stable throughout her hospital course and was able to breastfeed without difficulty. She was discharged home with her parents at 1 week of life, with scheduled follow-up in the pediatric ophthalmology clinic. She was seen at 3 months of age in clinic and was noted to blink to light, but could not fix and follow. The fundus examination was stable in both eyes.

## Discussion

Zika virus was originally identified in Uganda in 1947 in Rhesus monkeys [9], with outbreaks noted previously in Micronesia (2007) and French Polynesia (2013) [10]. ZKV is a single-stranded ribonucleic acid (RNA) Flavivirus spread by the *Aedes* mosquito or by sexual transmission. The *Aedes* mosquito is the same vector in the transmission cycle of dengue virus, chikungunya virus, and yellow fever. Zika is reported in countries with low altitude and close to sea level, as the *Aedes* mosquito cannot live above 6,500 feet [11]. Areas most at risk include Central America, South America, central Africa, Southeast Asia, and the Pacific islands. In the United States, the states with the highest incidence of symptomatic cases include New York, Texas, Florida, and California [2].

In adults, acute infection with ZKV can result in flu-like symptoms such as fever, conjunctivitis, maculopapular rash, headache, and joint pain that can last days to weeks [11]. However, only 20% of adults are symptomatic, and therefore the majority of adults with acute Zika infection are asymptomatic. Additionally, increase in rates of Guillain-Barré syndrome has been documented during ZKV outbreaks [12]. ZKV infection in the adult is diagnosed by blood or urine immunoglobulin M (IgM), Trioplex real-time reverse transcriptase-PCR (Trioplex rRT-PCR), or enzyme-linked immunosorbent assay (ELISA) [11]. The Center for Disease Control (CDC) developed the Trioplex-rRT-PCR to qualitatively detect and differentiate ZKV RNA from two other viruses, dengue, and chikungunya, which share the *Aedes* mosquito vector. The Trioplex PCR can be used in serum, whole blood, urine, amniotic fluid, and cerebrospinal fluid. By design, the Trioplex rRT-PCR minimizes likelihood of false positives due to low risk of cross-reactivity of test components [11].

In October of 2015, the Brazilian Ministry of Health reported a 20-fold increase in the number of infants with microcephaly in Northeast Brazil (99.7 cases per 100,000 live births in 2015 vs. 5.5–5.7 cases per 100,000 live births from 2000 to 2010) [5]. This increase in the incidence of microcephaly was linked to ZKV, secondary to transplacental infection from mother to fetus [11].

Studies have suggested that ZKV demonstrates neurotropism and preferentially infects undifferentiated neurons over differentiated neurons, explaining why neurological complications in the fetus are devastating whereas neurological complications in adults are rarer [13, 14]. Damage to neurons in early development leads to decreased number of neurons and can result in a smaller than normal brain with absence of gyri [15]. ZKV viral DNA and antigens have been detected via reverse-transcriptase PCR in formalin-fixed paraffin-embedded brain tissue of deceased infants with CZS and placental tissues of women with acute Zika infection during pregnancy that resulted in spontaneous abortions. Additionally, immunohistochemistry has identified ZKV in neural cells and areas of brain tissue with calcifications [14]. *In vitro* studies with human retinal cells show that the cells lining the blood-retinal barrier (BRB), the retinal endothelium, and the retinal pigment epithelium (RPE) are highly susceptible to ZKV-induced cell death compared to photoreceptor cells [16]. An *in vivo* study in a mouse model has shown that direct inoculation of ZKV in the eyes of adult mice results in chorioretinal atrophy and RPE mottling [16], similar to the changes seen in infants with congenital Zika infection. ZKV has also been identified in the optic chiasm, suprachiasmatic nucleus, lateral geniculate nucleus, and superior colliculus in a mouse model [17].

CZS is described as a pattern of birth defects seen in infants who have been infected with ZKV typically in the first or second trimester of pregnancy. The constellation of findings typically includes severe microcephaly due to arrested head growth, brain atrophy and ventriculomegaly, characteristic ocular findings, joint contractures, hearing loss, and hypertonia [5, 18, 19]. Microcephaly is defined as head circumference (HC) at birth > 2 standard deviations below the mean for gestational age and sex per the Fetal International and Newborn Growth Consortium for the 21^st^ Century [20]. Additionally, microcephaly associated with CZS can occur after birth, with the slowing of head growth in infancy. In 2017, the US Zika Pregnancy Registry Collaboration found that 11% of fetuses or infants presented with ZKV-associated birth defects among women with acute ZKV infection during first trimester of pregnancy [21].

Multiple studies from Brazil, Colombia, and Venezuela have described ophthalmic findings associated with CZS. The most commonly reported findings affect the posterior segment and include circumscribed macular chorioretinal atrophy, RPE mottling, and optic nerve abnormalities including hypoplasia, increased cup:disk ratio, atrophy, and pallor [6–8, 22]. The majority of findings are bilateral [7]. The pigmentary changes and chorioretinal atrophy show a predilection for the macula in the majority of described cases. Additionally, strabismus (esotropia and exotropia), nystagmus, and six reports of congenital glaucoma have been described in association with CZS [7, 22–24]. Risk factors statistically correlated with ocular involvement in infants with CZS include smaller head circumference at birth and symptomatic maternal infection in the first trimester of pregnancy [25].

A study by Ventura *et al.* [26] performed optical coherence tomography (OCT) on nine eyes of seven infants with positive cerebrospinal fluid IgM antibodies for ZKV and found that 69% of eyes had retinal abnormalities. The most common findings were interruption of the ellipsoid zone and RPE hyper reflectivity (100% of eyes), retinal thinning (89% of eyes), and choroidal thinning (78% of eyes). Four eyes demonstrated colobomatous-like excavation involving the neurosensory retina, RPE, and choroid (44% of eyes), which is similar to the findings seen in our patient. The infant in our case report also presented with bilateral choroidal thinning and absence of retina in the macula. The abnormalities seen on OCT in CZS infants localize to the inner layers of the neurosensory retina, and to the choroid in more severe cases. These findings highlight the neurotropism of ZKV, which was demonstrated by the *in vitro* human studies and *in vivo* animal studies mentioned previously, [13–16] and also the devastating effect the virus has on CNS and retinal tissues.

CZS shares many similar characteristics with other congenital infections such as rubella and toxoplasmosis such as microcephaly and hearing loss. Additionally, colobomatous-like lesions in the fundus have been described in association with congenital toxoplasmosis [27, 28]. However, differentiating factors of CZS include severe microcephaly with craniofacial disproportion, small brain size associated with thin cerebral cortex and microcalcifications, circumscribed macular atrophy and retinal pigment epithelium mottling, congenital joint contractures, and early hypertonia. The ophthalmic findings are specific enough to CZS, particularly macular atrophy, that fundus examination may help diagnose CZS in infants presenting without microcephaly. Additionally, CZS typically does not present with vitritis, chorioretinitis, or hyperpigmented scarring, distinguishing itself from congenital toxoplasmosis.

Given that the majority of women infected with ZKV in early pregnancy will be asymptomatic, whether or not to screen for ZKV should be determined largely by geographic or travel risk factors [21]. Dr. Tom Frieden, the director of the CDC revealed that the lifetime incremental cost of raising a child with CZS in the United States could cost anywhere from USD 1 to USD 10 million [29]. This staggering number is not a surprise given the severe neurologic impairment of these infants. A study of 31 infants with ELISA-proven CZS demonstrated all children demonstrated some degree of visual impairment. Sixty-five percent of infants did not respond to the Hiding Heidi test, a low-contrast “face” test, which evaluates the ability to detect objects with low contrast. Furthermore, 97% of infants were not able to perform at least one of the expected visual developmental milestones expected for their age group. For example, 26% of babies could not make eye contact and 52% had no social smile at 6 to 8 weeks of age. At 3 months of age, 83.9% of infants had no regard for hands, and at 5–6 months of age, 63% of infants could not perform a goal-directed reach, 74% did not use vision to perform reaching, and 93% could not bring their hands to midline [24]. The CNS and ocular abnormalities seen in CZS interfere with development of binocular vision and stereopsis in these infants, and all infants with CZS will require visual rehabilitation to achieve a functional level of vision.

## Conclusions

The role of the ophthalmologist is crucial in the care of infants with CZS. Additionally, all infants exposed to ZKV *in utero* may present with abnormal visual function, even if born without microcephaly or ocular abnormalities. The American Academy of Pediatrics and CDC recommends dilated eye examination within 1 month of life for every infant in which CZS is suspected [30]. Furthermore, the severe ophthalmologic impairment of these infants, including loss of stereopsis and the development of binocular vision, will require involvement of a low vision specialist. The needs of infants affected by CZS are complex and will require coordinated care through a multidisciplinary healthcare team, of which the ophthalmologist is an important component.

## Abbreviations

BRB: Blood-retinal barrier
CDC: Centers for Disease Control and Prevention
CNS: Central nervous system
CZS: Congenital Zika syndrome
ELISA: Enzyme-linked immunosorbent assay
HC: Head circumference
HIV: Human immunodeficiency virus
MRI: Magnetic resonance imaging
OCT: Ocular coherence tomography
RNA: Ribonucleic acid
RPE: Retinal pigment epithelium
Trioplex rRT-PCR: Trioplex real-time reverse transcriptase polymerase chain reaction
ZKV: Zika virus
